# A Meta-Synthesis of Review Studies on Wood–Polymer Composites: Mapping the Current Research Landscape

**DOI:** 10.3390/polym18010063

**Published:** 2025-12-25

**Authors:** Marius Nicolae Baba, Mirela Camelia Baba

**Affiliations:** 1 Department of Mechanical Engineering, Faculty of Mechanical Engineering, Transilvania University of Brașov, Eroilor Bvd. 29, 500036 Brașov, Romania; 2 Department of Finance, Accounting and Economic Theory, Faculty of Economic Sciences and Business Administration, Transilvania University of Brașov, Eroilor Bvd. 29, 500036 Brașov, Romania

**Keywords:** wood–polymer composites (WPCs), CiteSpace, co-citation network, cluster analysis, natural language processing, research landscape mapping, citation burst detection, bibliometric visualization, knowledge domain evolution

## Abstract

Wood–polymer composites (WPCs) consistently garner considerable attention owing to their material versatility and sustainability, resulting in numerous review studies across diverse disciplines. Nonetheless, since a comprehensive synthesis that consolidates these disparate reviews is lacking, this study performs a meta-synthesis of review articles focused on WPCs employing a science-mapping approach enhanced by CiteSpace software. A systematic search of the Web of Science Core Collection (last updated in June 2025) was conducted, yielding 51 review-type articles selected using PRISMA screening guidelines. Network-based co-citation, clustering, and keyword analyses reveal that recent WPC research centers on three interconnected areas: (i) reinforcement and interfacial engineering, (ii) processing–structure–property relationships, and (iii) sustainability-focused design involving recycling, fire safety, thermal pretreatment, and PCM-based thermal management. Sixteen author/reference clusters and nine keyword clusters highlight well-defined knowledge communities on durability and fire safety, nano- and bio-based reinforcements, recycled and bioplastic matrices, and advanced manufacturing techniques such as co-extrusion, flat-pressing, 3D printing, and wood–polymer impregnation. Timeline and burst analyses show that mechanical performance remains the primary focus, while emerging areas include recycled/waste-derived polymers, cellulose micro- and nanofibers, moisture-resistant hybrids, and wood-based additive manufacturing for construction applications.

## 1. Introduction

Wood–polymer composites, commonly called WPCs, are composite materials mainly made of synthetic thermoplastic resins combined with wood fillers (WFs), such as fibers or flour. Sometimes, thermosetting resins and naturally sourced resin systems are also used [1,2]. These composites combine the performance and cost advantages of natural wood with a polymer matrix. They have rapidly developed over the past few decades, increasing their application in industries such as gardening and landscaping products, boat decking, outdoor furniture, packaging, and consumer goods. However, the main growth area worldwide remains the production of automotive interior trim components and prefabricated buildings [3,4,5]. In this context, advancements in WPC research are crucial for promoting a sustainable future by efficiently using plastic waste, protecting the environment, reducing pressure on timber resources, and enabling large-scale production of affordable materials [6].

The most utilized thermoplastics in WPCs are polyethylene (PE), polypropylene (PP), polyvinyl chloride (PVC), polystyrene (PS), and polyamide 6 (PA 6) [7,8,9,10]. Although virgin thermoplastic polymers are the most prevalent, the increase in plastic waste, especially in municipal solid waste (MSW), has significantly promoted post-consumer plastic recycling and its application in wood–plastic composites (WPCs) [11,12,13,14,15].

Pre-processing treatments are applied to WPC components to improve the interfacial properties between wood fibers and the polymer, thereby reducing the hydrophilic nature of the wood fibers [16,17,18,19]. Chemical treatments are common; however, they cause environmental harm [20,21,22]. In contrast, mechanical and thermal treatments have been suggested as environmentally friendly alternatives to chemical methods [23,24].

Current technologies for producing WPCs are highly automated and adaptable to various raw materials [25]. Injection molding [26,27,28], extrusion [29,30], and compression molding [31,32] are currently the most widely used technologies in WPC manufacturing. However, over the past decade, advanced additive manufacturing techniques have revolutionized the field, including deposition layer modeling [33] and laser sintering [34,35,36].

**Figure 1:**
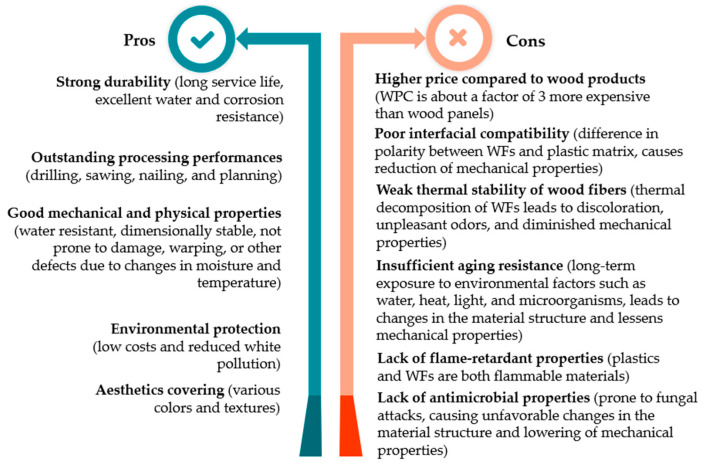
Pros and cons summary of WPCs.

After thoroughly reviewing most of the scientific publications cited in this section—articles, reviews, books, and conference papers—a pros and cons summary of WPCs was created, as shown in Figure 1. It is worth noting that despite their advantages, broad market potential, and numerous application opportunities, the development of WPCs is limited by several challenges.

As interest in recycled plastics and wood waste has grown in recent years, numerous research studies and review articles have been published that explore the features and uses of WPCs. The most recent and relevant ones are briefly summarized here. A review published by Wang et al. [37] examines research on co-extruded wood–plastic composites (Co-WPCs), focusing on their mechanical properties, moisture absorption, weathering resistance, flame resistance, and dimensional stability. Elsheikh et al. [38] thoroughly reviewed the manufacturing methods of WPCs, including related pre-processing and post-processing technologies. They discuss the three main pre-processing approaches—chemical, mechanical, and thermal treatments. Production processes for traditional WPCs covered include extrusion, injection molding, compression molding, stir casting, and hot-press procedures. Advanced methods such as selective laser sintering are also briefly addressed. The recycling of wood–plastic composites is discussed, along with proposals for eco-friendly evaluation. Bingyu et al. [39] examined raw materials and processing technologies for WPCs, along with the factors influencing their flexural properties. They assert that mechanical strength can be enhanced through surface pretreatments and the integration of compatibility and coupling agents. Zhang et al. [40] highlighted the crucial role of interfacial compatibility in determining the antibacterial effectiveness of WPCs and detailed common antimicrobial agents and their modes of action. Their goal is to improve understanding of the design and development of functional antibacterial WPCs. Ramli et al. [41] reviewed the fundamentals of WPCs made from polymer wastes and lignocellulosic fibers (LCF), including definitions, classifications, raw materials, additives, and properties. Mital’ová et al. [42] presented a concise overview of the history, composition, and mechanical properties of WPCs, with particular emphasis on the significance of wood as a lignocellulosic fibrous component. In a study conducted by Morchid et al. [43], the compatibility of various phase change materials (PCMs) with WPCs and their influence on mechanical properties were evaluated. They argue that the inclusion of PCM, more so at elevated levels, can be a solution for improved regulation of temperature and enhanced energy efficiency, albeit it can also present compatibility concerns, decreased thermal conductivity compared to the matrix polymer and wood fibers, adverse mechanical properties, migration and leaching of PCM over time, increased cost of production, and difficulty in uniform distribution. Zheng et al. [44] conducted a systematic review of additive manufacturing (AM) technologies, including 3D printing of contemporary WPCs. Their work also covers traditional manufacturing methods, pretreatment techniques for AM-produced WPCs, various AM processes suitable for WPCs, their applications, and future outlooks.

In addition to being bio-based and environmentally sustainable, wood–polymer composites are highly versatile and suitable for a wide range of applications. Chatterjee et al. [45] emphasized the importance of WPCs from the broader perspective of advocates for sustainable formaldehyde-free products. Schwarzkopf and Burnard [6] described the structural and non-structural functions of lignocellulosic and engineered wood panels. Schwarzkopf and Burnard [6] outlined the performance characteristics of WPCs and highlighted the environmental benefits of wood and plastic alternatives, which they identified as the main reasons for the widespread use of WPCs in the construction, automotive, and consumer product industries. Xu et al. [46] evaluated the effectiveness of WPCs in municipal engineering, landscape design, and building infrastructure, focusing on their mechanical strength, biological durability, flame-retardant properties, and applications. Supporting this, Hasanin et al. [47] demonstrated the potential of agricultural waste-based WPCs for eco-friendly, biodegradable packaging by enhancing fiber–matrix compatibility through fungal treatment. Zor et al. [24] incorporated nanotechnology, noting that nanomaterials embedded in WPCs can improve structural integrity, thereby opening up new possibilities for advanced manufacturing and environmentally friendly product development. Shrestha et al. [48] were among the first to explore the biomedical potential of WPC, partly motivated by the structural similarities of biological tissues, especially the potential of lignocellulosic bio-nanocomposites for drug delivery, medical diagnostics, wound healing, and bone regeneration. This wide range of applications—covering construction, transportation, biomedicine, and new materials—demonstrates the growing potential of WPCs as sustainable, high-performance alternatives across both traditional and emerging industries.

Although the reviews outlined above make significant contributions to the scientific literature by examining, summarizing, and synthesizing the current research status on wood–polymer composites (WPCs), it is widely recognized that such qualitative surveys have several inherent limitations. These include limited time coverage, a restricted ability to process large volumes of data, and, importantly, the influence of subjective judgment in selecting and interpreting sources [49]. Additionally, they often fail to provide a comprehensive, system-level overview of the broader research landscape, including collaboration patterns, thematic development, and emerging areas within the field.

To overcome these limitations and achieve a more objective, scalable, and comprehensive understanding of the research landscape, bibliometric analysis provides a valuable alternative. As a quantitative method that uses publication and citation metadata, bibliometric analysis allows for the investigation of scientific structures, outputs, and interactions across an entire field [50].

Unlike traditional narrative reviews, which depend on manual searches and subjective interpretation, bibliometric reviews use systematic mapping and statistical methods applied to large datasets. Researchers can organize and analyze complex bibliographic data with greater accuracy and reproducibility, revealing patterns through innovative data processing and visualization techniques. These methods help identify key data points such as main contributors and their publications, collaborative research networks, and emerging specialized areas [51]. Such insights can then be used to evaluate bibliographic data about authors, institutions, countries, and journals to determine their academic output, influence, and collaborative links [52]. As a result, bibliometric studies are valuable for researchers, offering a basis for evaluating academic work within a discipline or related fields and for discovering potential gaps or isolated research areas in the literature, regardless of the researcher’s expertise. In this context, the article by Ribeiro et al. [53] is relevant, as it provides a systematic review of the use of post-consumer plastics to produce WPCs for building components. Initially, 3111 articles were selected from academic databases using a specific search protocol. After applying strict exclusion criteria, 15 relevant studies on plastic waste composites were identified. These studies primarily focus on incorporating post-consumer polymeric materials, particularly polypropylene and high-density polyethylene, along with biogenic fillers, to enhance the mechanical properties of the composites and reduce reliance on virgin materials.

Nonetheless, the growing number of review studies—regardless of their form or scope—has unintentionally led to fragmentation and redundancy in the literature. These studies often focus on specific subfields or use diverse methods and terminology, leading to overlaps, inconsistencies, or insights that remain isolated. As a result, researchers, practitioners, and policymakers face significant challenges in developing a clear understanding of the field under investigation. In this context, a comprehensive meta-synthesis of existing review studies is crucial, as it offers a more advanced way to integrate findings beyond just summarizing individual reviews. That is, such a meta-synthesis systematically combines and consolidates the results to give a more complete picture of the research area. Essentially, it is both essential and necessary because it brings together scattered knowledge, assesses the progress of key research themes, and highlights critical areas that are either underexplored or in the early stages of development.

Consequently, this meta-synthesis review aims to systematically map and analyze the intellectual and thematic landscape of scholarly reviews on WPCs. The analysis relies on CiteSpace’s structural and temporal capabilities, a well-known science-mapping and visual analytics tool that reveals key patterns and structures in the scientific literature. Unlike more general bibliometric tools such as Bibliometrix or VOSviewer, CiteSpace can identify turning points in ideas, citation bursts, and structural changes in growing knowledge areas—features that are beneficial for synthesizing a fragmented collection of review studies [54,55]. For this reason, the present study uses CiteSpace to explore co-citation relationships, thematic clusters, influential authors, emerging topics, and the temporal development of the review literature on WPCs. The method provides a detailed, multidimensional view of the research landscape, enabling the identification of core knowledge areas, leading contributors, and emerging themes. However, it is worth mentioning that this review does not assess intervention effects, as the outcomes are based on network properties such as co-citation frequency, cluster structure, and burst strength.

Carrying out the meta-analytical approach described above, the study was guided by the following research questions (RQ):

Co-Citation Analysis of References—RQ1: *Which scholarly references are most frequently co-cited in WPC review literature, and what insights does their co-citation network provide about the field’s conceptual foundations?*

Clustering of Co-Cited Literature—RQ2: *What are the main thematic clusters formed through co-citation of literature, and how do these clusters define the intellectual structure and knowledge areas of WPC research?*

Co-Citation Patterns of Influential Authors—RQ3: *Who are the most influential authors in the review literature on WPCs as determined by author co-citation analysis, and what structural positions do they occupy in shaping the development of the field?*

Keyword Co-Occurrence Analysis—RQ4: *What are the primary and emerging research themes identified through keyword co-occurrence networks, and in what ways do they influence the current focus of WPC review studies?*

Temporal Timeline Visualization—RQ5: *How have the main themes and clusters in WPC research evolved over time, and what chronological patterns can be identified from the timeline visualization of thematic progress?*

The rest of this paper is organized as follows: Section 2 describes the data collection process, the criteria used to select review articles on WPCs from the WoS Core Collection, and outlines the methodological workflow for CiteSpace. Section 3 combines the meta-synthesis and examines Co-Citation of References, Co-Cited Literature, Author Co-Citation, Keyword Co-Occurrence, and Timeline Visualizations, presenting the results, interpretation, and discussion of each dimension across the five components. Finally, Section 4 summarizes the conclusions.

## 2. Data Source and Methodology

**Figure 2:**
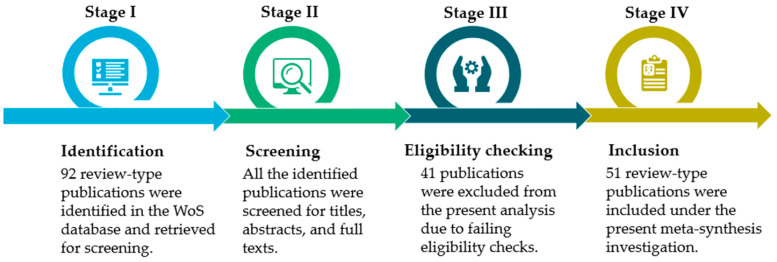
PRISMA flowchart applied to current research.

The rigorous, transparent methodology used in this paper to oversee the data collection process follows the Preferred Reporting Items for Systematic Reviews and Meta-Analyses (PRISMA) standards [56] (see Table S1 for the specific PRISMA checklist). Figure 2 presents the PRISMA flow chart used in this study, supporting the systematic identification, screening, and inclusion of studies in an organized, reproducible manner, which is essential for any high-quality bibliometric analysis. The relevant literature for this meta-synthesis was gathered using a customized, comprehensive search method developed explicitly for the WoS Core Collection database. The construction of this search query is outlined in Table 1, organized into three conceptual blocks, and connected with logical “AND” operators to refine search results and increase the relevance of the retrieved articles.

The initial stage of the systematic search involved finding literature on wood–polymer composites using a limited set of keywords. This includes both hyphenated and non-hyphenated versions, such as “wood-plastic”, “wood plastic”, “wood-polymer”, and “wood polymer”, with an asterisk indicating any word endings such as “composite”, “composites”, and similar variants to ensure that the search captures a wide range of articles with different phrasing.

The second search term, “composite”, refined the search to focus on studies concerning composite materials, thereby eliminating extraneous results associated with alternative applications of the terms “wood” and “polymer”.

The third part of the search concentrated on review-type studies, using the descriptors “review”, “literature review”, “systematic review”, “scoping review”, “state of the art”, and “bibliometric analysis”. It aimed to make sure that the search was limited to publications that used review-based methods to compile knowledge within the discipline.

By integrating these three groups of terms within the Topic field (TS), which includes titles, abstracts, and author keywords, the strategy was designed to optimize both comprehensiveness and relevance. The search query was completed by June 2025 without any restrictions on publication date, allowing the retrieval of both pioneering early studies and the latest advancements in the field. No language limitations were applied during the initial search to ensure the broadest possible inclusion of relevant publications. However, because the analysis relied on information from titles, abstracts, and keywords, only English-language abstracts directly related to the research focus were selected for further analysis. No formal risk-of-bias tool was used, since the study aims to map the intellectual structure of review articles rather than quantitatively synthesize effect estimates. Methodological limitations are discussed qualitatively in the next section. This review was not prospectively registered, and no separate protocol was prepared.

Based on the search criteria mentioned above, the identification phase produced an initial set of 92 review-type publications. Data extraction from the included articles was performed using a predefined extraction scheme. Then, the bibliographic data were exported from the WoS Core Collection database in plain text format, including full records and cited references, to facilitate further CiteSpace analysis. Two reviewers independently checked the exported records (authors, titles, journals, years, document types, cited references, and keywords) for consistency, and any discrepancies were resolved by consensus.

The screening process consisted of two stages to ensure methodological rigor and thematic consistency. First, titles and abstracts were examined to determine if the publications addressed WPCs. In the second stage, full texts were reviewed to evaluate each publication’s relevance and verify its eligibility for inclusion in this meta-synthesis. Two reviewers independently assessed eligibility, and any disagreements were resolved through discussion until consensus was reached.

During the eligibility assessment, clearly defined inclusion and exclusion criteria were used. As mentioned earlier, publications were included if they were peer-reviewed articles focusing on surveys or advanced reviews, such as narrative reviews, systematic reviews, scoping reviews, and bibliometric analyses. Each study also had to explicitly focus on the development, characterization, performance, application, or sustainability of wood–plastic composites. In this context, the following exclusion criteria were applied:

- Non-review publications: Many records were excluded because they were original research articles rather than reviews. These works include experimental investigations, material formulations, mechanical testing, or case-specific designs instead of synthesizing the existing literature.
- Publications that lack focus on wood–polymer composites as the central theme: Some articles mentioned WPCs only peripherally or within the broader material categories (e.g., biocomposites, green materials, natural fiber composites, cellulosic composites or nanocomposites, plastic and wood recycling logistics; pallets and packaging systems; additive manufacturing) without making them the primary subject of analysis.

At the end of the inclusion phase, 51 scientific publications were retained, and the resulting dataset was thoroughly preprocessed to ensure consistency and compatibility with CiteSpace software for subsequent bibliometric mapping. Special emphasis was placed on formatting cited references (CR fields) and addressing inconsistencies, such as periods separating initials, surnames written entirely in uppercase, complete forename formats, and anonymous entries. Because these issues caused fragmented citation records and inaccurate representations of co-citation relationships, all entries within the dataset were carefully examined and standardized to ensure uniform formatting, thereby improving the accuracy and reliability of the co-citation and author network visualizations.

Furthermore, to ensure the accuracy of citation records and author identification in the bibliometric analysis, attention was paid to maintaining consistency in published author names, particularly for individuals with identical surnames. Such inconsistencies, if ignored, could lead to fragmented citation networks and underreporting of an author’s contributions. Therefore, all these variations were manually checked and combined, ensuring that each author’s publication record was treated as a single, unified identity in the co-citation and collaboration analyses in CiteSpace.

**Figure 3:**
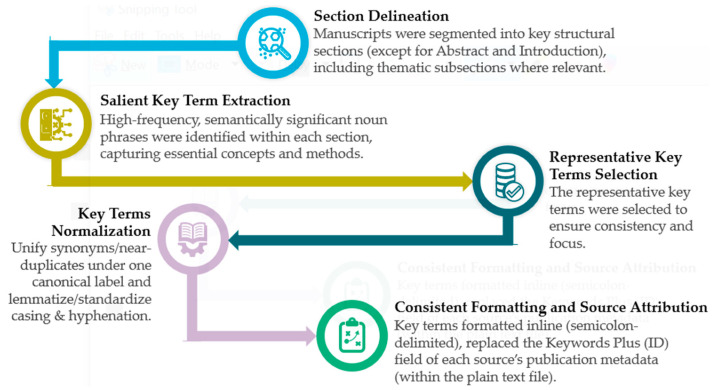
The workflow of the MFKTM approach.

A methodological challenge faced during data preparation was the limited semantic accuracy and inconsistent relevance of Keywords Plus for thematic mapping. As Clarivate Analytics notes [57], Keywords Plus are derived from terms frequently found in the titles of an article’s cited references, rather than the article itself. These are automatically generated using a proprietary algorithm and often emphasize interdisciplinary breadth over topical precision. Although such terms can help identify citation trends, their effectiveness in capturing the actual content of review articles is relatively limited [58]. Indeed, inspection showed that Keywords Plus and, in many cases, Author Keywords did not consistently reflect the articles’ core contributions or themes. Consequently, to improve topical clarity and analytical accuracy, this research introduces a Methodological Framework for Key Terms Mapping (MFKTM), illustrated in Figure 3. This method systematically extracts and classifies relevant phrases from different sections of each review examined in this study, enabling a more precise, context-aware theme analysis.

As noted in the introduction, the bibliometric analysis was performed using CiteSpace (version 6.4.R.2), a popular software tool for visualizing and analyzing the structure and temporal patterns of scientific literature. Additionally, it allows the creation of various network-based visualizations, such as reference co-citation networks, keyword co-occurrence maps, and timeline trend analyses.

## 3. Results and Discussion

Guided by the five interconnected research questions presented in the introduction, this section summarizes and discusses key findings from the meta-analytical mapping of the literature on wood–plastic composites. Each subsection concentrates on a distinct analytical topic, including reference co-citation analysis, clustering of co-cited literature, author co-citation patterns, keyword co-occurrence analysis, and timeline visualization. These features provide a comprehensive view of WPC research’s conceptual foundations, thematic structure, influential authors, key topics, and chronological development.

### 3.1. Co-Citation Analysis of References—RQ1

**Figure 4:**
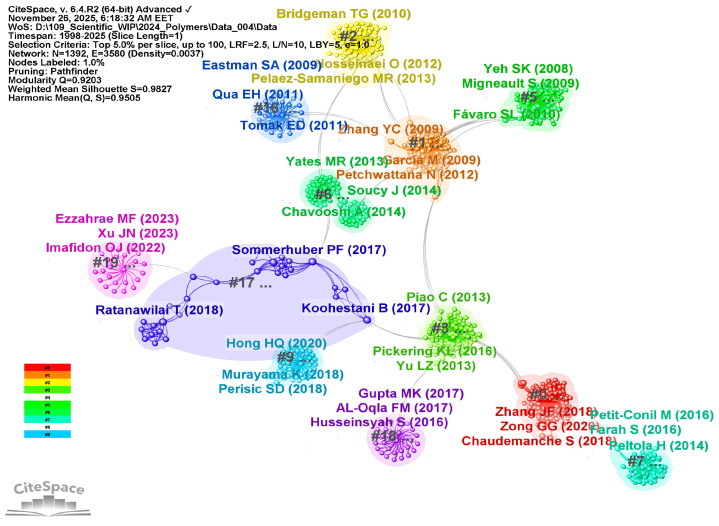
CiteSpace visualization of reference co-citation “by-cluster” in the WPC literature.

**Figure 5:**
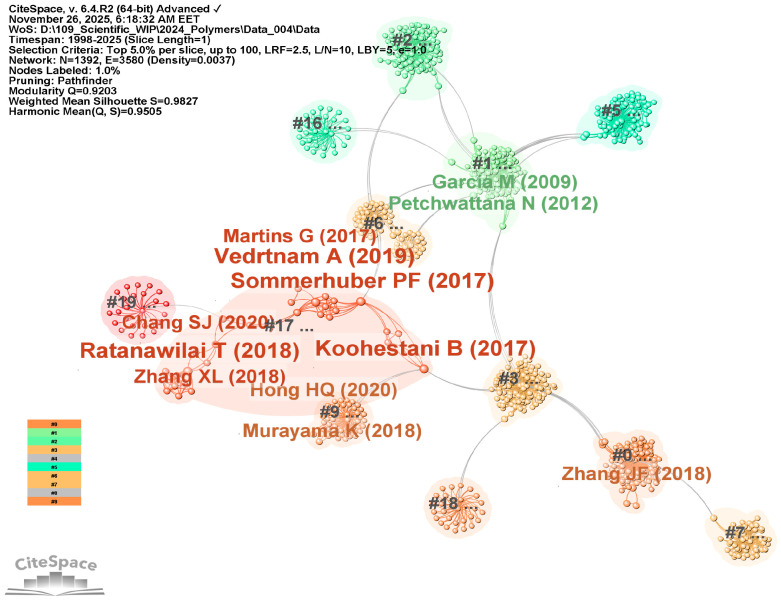
CiteSpace visualization of reference co-citation “by-citation” in the WPC literature.

To clarify the intellectual foundations shaping current WPC research, a co-citation analysis of references was conducted using CiteSpace. To identify influential works frequently cited together, the node type “Cited References” was selected. The resulting network produced 12 major co-citation clusters, as shown in Figure 4, through a detailed by-cluster visualization that illustrates how groups of references form cohesive thematic areas. Node colors indicate cluster identity, node sizes represent citation frequency, and links between clusters show intellectual connections. For consistency, Figure 5 presents a by-citation visualization highlighting the most influential authors whose work underpins these domains. In this visualization, larger nodes denote a higher citation influence, emphasizing authors whose contributions unify multiple thematic areas.

Aiming to structure the discussion in a conceptually aligned manner, the co-cited references were interpreted according to three research directions intended to unify the 12 computational clusters, as reported in Table 2, namely (1) material reinforcement and interfacial engineering, (2) processing behavior and structure–property relationships, and (3) sustainability, recycling, fire safety, and thermal enhancement. Such an approach enables a clear understanding of how frequently co-cited references collectively define the field’s scientific backbone.

***Material reinforcement and interfacial engineering*** constitute one of the most mature and conceptually influential pillars in WPC research. This research area consolidates a wide array of studies investigating how the intrinsic properties of wood-derived reinforcements—ranging from cellulose particles to refined fibers—and their interfacial compatibility with polymer matrices influence the mechanical, physical, and long-term performance of WPC systems. The co-citation network reveals these foundational works, constituting a dense, interconnected core indicative of robust intellectual continuity across topics such as load transfer, fiber/matrix adhesion, particle morphology, and surface modification techniques.

A central contribution in this domain is provided by Jian et al. [39], whose comprehensive review on the flexural properties of WPCs has become one of the most frequently co-cited works in the field. Their work outlines how the interplay between raw material selection, processing conditions, and interfacial treatments governs reinforcement effectiveness. Based on a comprehensive evaluation of coupling agents (specifically maleic anhydride-based systems), fiber surface pretreatments, and geometric parameters, including fiber aspect ratio, the authors determine that mechanical performance (i.e., flexural strength and modulus) is maximized through the practical engineering of interfacial adhesion and load-transfer mechanisms.

Hubbe and Grigsby [59] provide a detailed analysis of how particle size affects cellulose-based reinforcements. Their review, which covers micro- to nano-scale additives, discusses particle rigidity, density, dispersion, and interfacial compatibility. As they mention, although nanocellulose has a very high surface area, improved adhesion often requires a larger amount of compatibilizer, and overall gains in mechanical properties may not always be seen. This work has guided future research, demonstrating that reducing particle size alone is insufficient; instead, optimizing interfacial chemistry and morphology requires a targeted strategy.

Building on the reinforcement design, Zhang et al. [60] provide influential evidence by demonstrating the benefits of natural fiber hybridization, particularly the incorporation of flax fibers into wood flour/polyethylene composites. Their experimental results show substantial gains in flexural modulus, impact strength, and creep resistance, demonstrating that careful fiber selection can synergistically enhance performance without altering the polymer content. These findings inform many later strategies in hybrid reinforcement and fiber engineering, especially when balancing stiffness–toughness trade-offs.

Interfacial engineering is further intensified by Anbupalani et al. [61], who compile and evaluate the growing body of research on coupling agents for natural-fiber composites. Their review clarifies how chemical compatibilizers (i.e., silanes, maleic anhydride derivatives, and isocyanates) mediate adhesion across inherently incompatible hydrophilic–hydrophobic interfaces. The authors provide mechanistic and application-level insights into how these agents modify fiber surfaces, reduce interfacial defects, and improve stress transmission, thereby establishing one of the conceptual cornerstones of modern WPC formulation strategies.

Finally, seminal contributions from Fang et al. [62] and Piao et al. [63] enrich the understanding of surface modification to enhance composite stability. The former demonstrates the effectiveness of silane treatments in promoting covalent bonding between the wood and polymer phases, resulting in notable improvements in shear strength and water uptake resistance. At the same time, the latter extends this line of work by showing that siliconate treatments reduce water sorption and volumetric swelling while improving filler–matrix compatibility. Both these studies have established that surface-chemistry modifications are a reliable route to durable, dimensionally stable composites, especially for moisture-sensitive applications.

To a great degree, the publications outlined above constitute the fundamental framework of the interfacial paradigm in WPC research. Their co-citation underscores the significance of interfacial adhesion, particle morphology, and compatibilization in enhancing material performance. Furthermore, the cohesive structure of these works underscores this theme as a prospective foundation for future developments in both conventional and innovative wood–plastic composites.

The second research pillar examines how ***processing conditions, fiber preparation, rheological properties, and the development of composite microstructures*** collectively influence the performance spectrum of WPCs. The existing co-citation network demonstrates that these studies constitute a cohesive body of knowledge, illustrating collective endeavors to comprehend how minor variations in processing—such as fiber size, blending technique, screw speed, and the application of interfacial modifiers—can lead to notable differences in mechanical properties, dimensional stability, and durability. This body of work effectively connects engineering principles with manufacturing practices, emphasizing that the performance of WPCs is fundamentally linked to their processing history.

Migneault et al. [64] is a key reference in this research area, as their contribution is among the most comprehensive and influential when comparing extrusion and injection molding of WPCs. It has been shown that the mechanical properties of injection-molded composites are enhanced, while their water absorption is reduced. Better fiber integration, fewer processing cavities, and improved compaction explained this. They also recognized the fiber aspect ratio as a critical parameter, noting that longer fibers are more effective but also induce greater anisotropy and affect moisture diffusivity. This significant research demonstrated that composites are structurally and functionally engineered during processing, necessitating a customized approach to processing decisions to fulfill end-use requirements.

The parallel studies conducted by Yeh and Gupta [65] further advanced the process–structure–property approach by demonstrating that optimizing processing alone (in the absence of chemical compatibilizers) can markedly reduce water absorption. The controlled adjustment of screw rotation speed and melt residence time revealed that changes in melt mixing more effectively improve fiber wetting, minimize internal voids, and therefore shorten water diffusion pathways. Research by Fávaro et al. [66] further clarified the relationship among fiber treatment, chemical structure, and composite structure. Through the analysis of the mercerization and acetylation of rice husk fibers, a correlation was established between fiber surface chemistry and alterations in interfacial adhesion, matrix infiltration, and ensuing mechanical performance. Their research revealed that specific chemical alterations influence processing by modifying melt rheology, dispersion quality, and interfacial load transfer efficiency, underscoring the essential relationship between fiber preparation and processing behavior.

The rheology-focused studies by Murayama et al. [67], Hong et al. [68], and Perisic et al. [69] form another pivotal part of this section. Together, these works improved understanding of how compatibilizers, interfacial modifiers, and hybrid reinforcements influence melt behavior, thereby affecting shear viscosity, flow instability thresholds, and fiber–matrix interactions during compounding. These studies agree that rheological tailoring is key to achieving composite uniformity, structural stability, and long-term performance. Lastly, the contributions of Peltola et al. [70] and Pickering et al. [71] provide valuable comparisons of how fiber type, polymer matrix choice, and dispersion mechanisms control the development of composite architecture during melt processing.

The high co-citation frequency of these seminal works underscores a consensus within the field: the transition from raw materials to optimal performance of WPCs is influenced by processing-induced structure, and innovations in rheology, fiber engineering, and melt-processing techniques that are essential for enhancing the reliability and multifunctionality of WPCs.

The third pillar of WPC research is an expanding domain that integrates sustainability-oriented material design, improved thermal stability, and exceptional fire performance, all of which correspond with future societal and industrial requirements. As environmental concerns grow and regulations change, WPC research has moved from a focus solely on performance-based materials engineering to a broader approach that includes ***life-cycle assessments, sustainable practices, and manufacturing that emphasizes functionality*** beyond just mechanical performance. The co-citation network shows that studies dealing with ecological efficiency, recyclability, thermal pretreatment, and advanced flame-retardant or thermally responsive features form a closely connected intellectual domain—one that supports the field’s long-term strategic growth.

A key element of this approach is the highly influential life-cycle assessment by Sommerhuber et al. [72], which remains the most frequently co-cited reference in the sustainability-focused part of the network. The authors provide clear evidence that wood–plastic composites (WPCs) made from secondary materials, especially recycled plastics and wood waste, have a notably better environmental profile than those made from virgin materials. The study indicates that addressing both upstream resource supply and downstream end-of-life recycling substantially mitigates environmental consequences and enhances circularity; however, existing market and legal frameworks primarily support incineration.

Ratanawilai and Taneerat [73] conducted pioneering research on the resistance of alternative polymer matrices to weathering and mechanical degradation, highlighting the superior weathering resistance of polystyrene and polypropylene, which retain greater mechanical properties than polyethylene, underscoring the significance of polymer selection in the design of WPCs. These findings strengthen the understanding of the sustainability benefits associated with the functional service life of durable products.

The ability of silane-functionalized mine tailing minerals to act as functional fillers in wood-plastic composites represents a new approach to sustainable development, as shown by the research of Koohestani et al. [74]. Their study revealed that small amounts (1–5%) of amine- and vinyl-modified mineral fillers improved the mechanical properties, thermal stability, and rheological response of WPCs.

The thermal pretreatment of wood is another principal focus of this area, as highlighted by Pelaez-Samaniego et al. [75] and Hosseinaei et al. [76]. Prior work focused on hemicellulose, and hot-water and steam pretreatments explain the key advantages in the manufacturing processes of WPCs: removal of hemicellulose, reduction of hygroscopicity, increased cellulose crystallinity, and improved resistance of WPCs to dimensional changes and microbial degradation. The latter work has built on this by extracting hemicellulose and removing some hydrophilic components, yielding additives with reduced water absorption, higher thermal stability, and improved interfacial adhesion. The WPCs produced are less prone to swelling and greater in tensile strength. The combination of these studies makes thermal pretreatment one of the simplest and most sustainable techniques for improving the value and broadening the applications of bio-composites.

Another key element of the sustainability-focused pillar is fire safety. The co-citation core, comprising Mokhena et al. [77], Renner et al. [78], and Eastman et al. [79], indicates that this research field is now gaining a foothold. In materials engineering, there is a desire to achieve an optimal balance between fire resistance and other mechanical attributes. Mokhena et al. review progress in flame-retardant WPCs and report ongoing difficulties in achieving the right balance between firmer flame resistance and weaker mechanical properties. Renner et al. broaden this perspective to consider flame retardancy in the study of WPCs and how various classes of fire retardants affect the composite’s combustion characteristics and microstructure, thereby improving its fabrication and use. The most original work of the four is that of Eastman et al., which, in a notable achievement, uses supercritical CO_2_ to assist in the infusion of silicone into wood, forming composite materials that exhibit high fire resistance and good mechanical properties. Such a promising approach combines durability and safety without sacrificing structural performance.

The works of Ezzahrae et al. [43], Xu et al. [80], and Imafidon and Ting [81] broadened the linkage between renewable materials and energy-efficient construction technologies, amid emerging concerns about the thermal energy storage of wood–plastic composites. Ezzahrae et al. described the prospective incorporation of phase change materials (PCMs) in wood–plastic composites (WPCs) and the substantial demand for passive thermal control in eco-friendly buildings. Xu et al. demonstrated that biomass-derived activated carbon is a promising carrier for polyethylene glycol-based phase change materials, enabling the synthesis of form-stable wood–plastic composites with high thermal storage and mechanical properties. Improved thermal storage and mechanical properties were obtained with polyethylene glycol-based phase change materials. Imafidon and Ting, through building-scale simulations, demonstrate the significant heat flux and energy savings possible with PCM-enhanced envelopes, while also improving the sustainability of WPC-based components.

The highly co-cited studies mentioned above clearly show a significant shift in WPC research toward materials that are not only high-performing but also thermally functional, environmentally sustainable, and resilient under high heat. Additionally, they indicate that sustainability—covering resource use, durability, fire safety, and thermal management—becomes an increasingly important factor in the development of new WPC technologies.

### 3.2. Clustering of Co-Cited Literature—RQ2

Unlike the co-citation analysis of references, the clustering of co-cited literature collects references into distinct thematic clusters. The aim of clustering extends beyond assessing the influence of individual references; it also aims to uncover the domain’s structural and conceptual organization. By consistently integrating co-cited references across various source publications, it facilitates the identification of prospective research directions, related subfields, and underlying intellectual networks.

**Figure 6:**
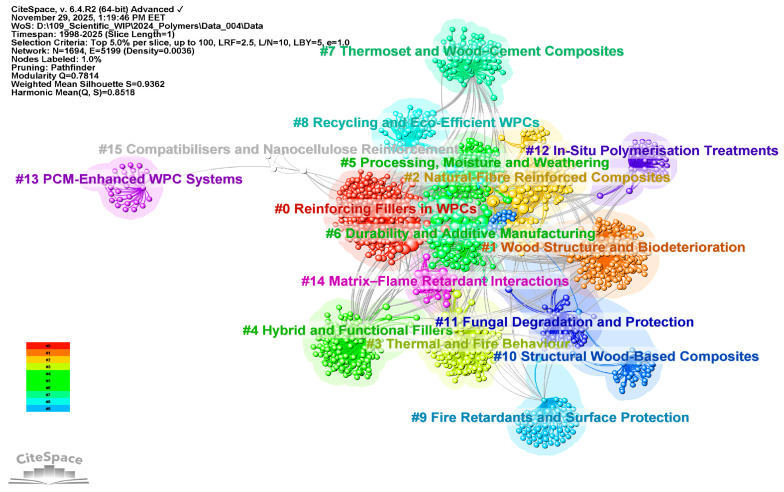
CiteSpace visualization of co-citation clusters of references in the WPC literature.

**Figure 7:**
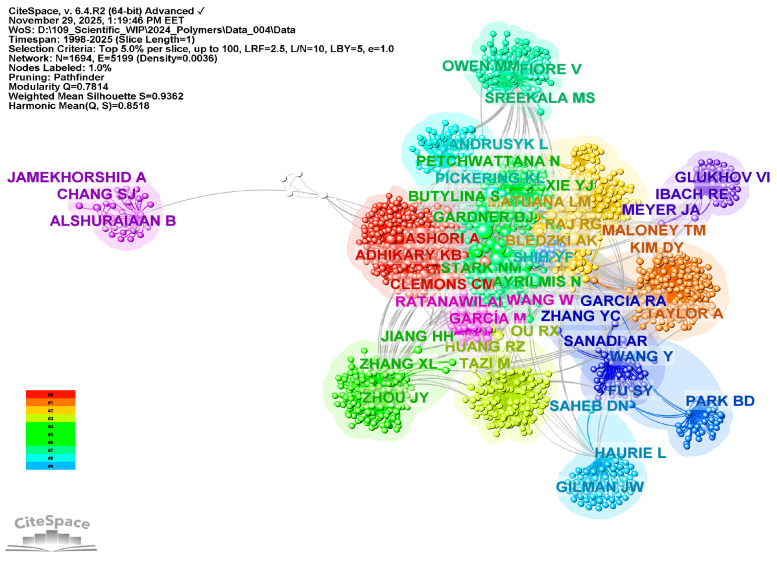
CiteSpace visualization “by-cluster” of references’ co-citation in the WPC literature.

The co-citation network was generated in CiteSpace using “cited author” as node type. A total of 16 major clusters, as shown in Figure 6, were identified and manually re-labeled following CiteSpace’s interpretative procedure. That is, inspecting the most frequently co-cited authors, their landmark publications, along with the title and abstract terms of highly cited reviews related to each cluster. Figure 7 overlays the most influential cited authors on these clusters, highlighting the intellectual communities that shape the actual structure of the WPC research field.

Cluster #0 is dominated by the work of Ashori and co-authors on WPCs and related composites formulated with bagasse, wheat straw, rice husk, municipal solid waste, waste timber railway sleepers, and microcrystalline cellulose, almost always in combination with polyolefins and coupling agents such as MAPE [82,83,84,85,86,87,88,89]. These studies systematically analyze how filler type, particle size, extractive removal, and compatibilizer content affect tensile, flexural, impact properties, and water absorption of PP/HDPE-based WPCs. Clemons et al. [90,91,92] and Adhikary et al. [93,94,95] extended this theme to recycled thermoplastics and waste streams, emphasizing the dimensional stability, long-term moisture uptake, and freeze–thaw durability of HDPE/PP–wood composites. Altogether, these works focus less on a single mechanism and more on formulation strategies—such as the choice of recycled polymers in line with agro-residues, and compatibilizers—intended to optimize global performance and sustainability of WPCs, which motivates the cluster title “Recycled and Agro-Filler WPCs”.

Cluster #1 is anchored to the research studies of Kim and collaborators on cellulose crystallite decomposition and surface acetylation of bacterial cellulose, which provide a fundamental description of how crystalline cellulose domains respond to heat and chemical modification [96,97]. These are complemented by the contributions of Taylor et al. [98,99,100] and Maloney et al. [101,102], which enframe WPCs and related engineered wood products in terms of industrial context, processing routes, and degradation mechanisms (e.g., primers on WPC technology, CLT/mass timber durability, and classic monographs on particleboard and composite manufacturing). On the whole, they map the conceptual and technological foundations of wood structure, thermal behavior, and engineered wood–polymer systems, rather than specific new formulations, so the label “Wood Structure and WPC Fundamentals” is appropriate.

Cluster #2 is dominated by a large body of work from Bledzki and co-workers that systematically compares softwood, abaca, jute, kenaf, and flax fibers as reinforcements in PP, PLA, PHBV, and related matrices, including acetylation, alkali treatment, and the use of MAH-PP for interfacial compatibilization [103,104,105,106,107,108,109,110,111,112]. The emphasis is clearly on mechanical performance (stiffness, strength, creep, impact) as a function of fiber species, fiber treatment, and fiber content, often with direct relevance to automotive applications (“Cars from bio-fibres”). Matuana et al. [113,114,115,116,117,118,119,120,121,122,123,124,125,126] and Raj et al. [127,128,129,130,131,132,133,134,135] extend this interfacial focus to PVC and polyolefin systems, investigating silanes, anhydrides, and isocyanates, as well as microcellular foaming in PVC/wood, PP/wood, and HDPE/wood composites. Across these works, the dominant co-citation signal concerns natural-fiber reinforcement and tailored fiber–matrix interfaces in thermoplastics, as reflected by its label “Natural-Fibre Reinforcement and Interfaces”.

Cluster #3 is centered on Huang et al.’s work on talc- and basalt-fiber-filled HDPE shells in co-extruded WPCs, quantifying changes in thermal expansion, mechanical modulus, and cone-calorimeter fire behavior as shell composition and filler content vary [136,137,138,139,140]. Ou et al.’s contributions on citric-acid-esterified wood flour, ionic liquids, and controlled removal of hemicellulose/lignin connect directly to melt rheology (torque, viscosity, storage/loss moduli) and dimensional stability in HDPE/wood blends [141,142,143,144,145]. Tazi and collaborators further develop rheological and thermophysical characterization (master curves, activation energies, relaxation times, viscoelastic behavior) alongside durability under moisture, fungal attack, and biodegradation [146,147,148,149]. Overall, this cluster is coherently focused on how structural modification and filler selection control thermal expansion, flow behavior, and flammability of WPCs, which justifies the label “Thermal, Rheological and Fire Behavior” as an accurate summary of its intellectual core.

Cluster #4 is influenced by the works of Zhang and collaborators, which explore nano-SiC-treated wheat straw fiber and flake-graphite-filled WPCs and link nano- and micro-scale fillers to crystallinity changes, enhanced stiffness, improved water resistance, and modified heat-transfer behavior in HDPE-based composites [150,151]. Furthermore, Jiang et al.’s studies on PVC–wood systems demonstrate that copper-amine treatments and hybrid glass-fiber/wood-flour reinforcement can tune surface energy, interfacial shear strength, and mechanical performance, while reviews summarize PVC/wood composites, coupling agents, and hybrid reinforcements with mica or glass [152,153,154]. The inclusion of antibacterial PLA systems modified with wood fiber/polydopamine/silver further highlights the formation of functionalized interphases (antibacterial and potentially conductive/thermally conductive) in polymer/wood systems [155]. The unifying theme is the use of nano- and hybrid inorganic fillers, along with interfacial chemistry, to impart new functionalities beyond simple stiffness gains, so the label “Functional Nanofilled and Hybrid WPCs” appropriately reflects the intellectual focus of this cluster.

Cluster #5 is driven by Xie et al.’s work on silane coupling agents and hydrophobation treatments of wood particles for PP composites, demonstrating how amino-alkylsiloxane co-oligomers and trialkoxysilanes simultaneously decrease water uptake and modify tensile, flexural, and impact behavior by altering the wood–polymer interphase [156,157]. A series of papers by Butylina and collaborators examines PP-based WPCs, including co-extruded composites with inorganic pigments and mineral fillers, and tracks accelerated and natural weathering, color change, tensile properties, microcracking, and freeze–thaw cycling [158,159,160,161,162,163,164]. Petchwattana et al. extend this line to PBS, HDPE, and PVC matrices, analyzing water-absorption kinetics, particle-size effects, natural weathering, and the recyclability of rice-hull and sawdust-based WPCs [165,166,167,168]. The research studies of Migneault and his co-workers add a complementary focus on fiber surface chemistry, fiber origin, proportion, and length, relating oxidized versus unoxidized carbon, residue-based fibers, and fiber aspect ratio to tensile strength, stiffness, water uptake, and swelling in HDPE WPCs produced by extrusion or injection molding [64,169,170,171]. The common denominator is the control of surface chemistry (silanes, pigments, minerals, fiber surfaces) to mitigate moisture uptake, color fading, and weather-induced damage in WPCs, which justifies the label “Surface Treatment and Weathering of WPCs” and explicitly links the issues of hydrophilization, pigmentation, and weathering.

Cluster #6 is dominated by the research corpus of Stark and collaborators, which addresses multiple facets of WPC durability and fire behavior: evaluation of additive-type fire retardants and heat-release response in wood–PE composites, co-extruded HDPE cap layers for color stability and moisture control, and detailed FTIR/XPS studies of weathered WPC surfaces that relate photodegradation and moisture uptake to losses in stiffness and strength [172,173,174,175,176,177,178,179,180,181,182,183,184]. In the same framework, it is worth noting that Stark’s broader chapter on wood-based composites situates WPCs within the wider family of panel and structural products and highlights durability requirements for outdoor use [185]. Gardner et al. contribute a 10-year field-exposure study in Chile along with an extensive technology overview that, together, emphasize the long-term biological, mechanical, and physical performance of WPCs and the emergence of additive manufacturing routes using wood-based fillers [186,187]. Ayrilmis and collaborators provide an extensive body of work on boron- and phosphate-based fire retardants, panel-pressed and thermally treated lignocellulosic composites, and 3D-printed wood/PLA filaments, systematically documenting the effects on dimensional stability, surface quality, fire response, and mechanical properties [188,189,190,191,192,193,194,195,196,197,198,199,200,201,202,203,204,205,206]. Turku et al.’s contributions focus on nanofillers and fire-retarded WPCs, WPCs manufactured from recycled plastic blends, the flammability and durability of recycled plastic-based composites, and nanocellulose-derived fire-retardant thin films and coatings, further linking weathering, recyclability, and fire safety to advanced WPC technologies [207,208,209,210,211,212,213,214,215,216,217]. Li and co-workers extend this perspective to glulam fire performance and to WPCs reinforced with bamboo charcoal or organic vermiculite. In their research papers, they provide a broad review of chemical treatments for natural fibers, linking interfacial modification to mechanical, thermal, and water-resistance behavior [218,219,220,221]. Overall, these references define a co-citation cluster organized around outdoor and hygrothermal durability, fire performance (including FR systems and flammability of recycled/nanostructured WPCs), and emerging manufacturing and recycling strategies such as co-extrusion, flat-pressing, 3D printing, and advanced coatings, which justifies the label “Durability, Fire and Manufacturing Routes”.

Cluster #7 is anchored to the research studies of Owen and collaborators, which are focused on developing composites from industrial plastic waste reinforced with recycled nylon fibers for structural paving tiles, and epoxy-coated kenaf fiber in RPET, demonstrating that thermoset surface coatings can stabilize natural fibers at processing temperatures above 200 °C and markedly improve thermo-mechanical performance [222,223]. Sreekala et al.’s classic work on oil palm fibers details acetylation, mercerization, latex coating, silane and isocyanate treatments, and their influence on water sorption, mechanical performance, and hybrid glass/oil-palm phenolic composites [224,225,226]. The contributions of Fiore and collaborators on the use of NaHCO_3_ and NaOH treatments of flax, jute, and kenaf fibers further underline the role of eco-friendly chemical modification in improving marine ageing resistance and composite strength [227,228]. Co-citation patterns thus converge on surface-treated natural fibers, often hybridized with glass or nylon, intended to withstand aggressive thermal and environmental exposures. This is the reason why the label “Hybrid Natural–Synthetic Fibre Composites” emphasizes hybrid natural–synthetic reinforcement and environmental resistance.

Cluster #8 builds upon extensive research by Pickering and collaborators on natural-fiber composites. It includes a wide range of factors related to the mechanical properties of optimized fiber and harakeke fiber of JP, along with detailed analyses of coupling agents and silane treatments used on radiata pine fiber in PP [71,229,230,231,232,233]. These results are complemented by Shih et al.’s work on biobased composite systems (pineapple leaf and recycled chopsticks in PLA/PBS; phytic-acid-modified wood flour and diatomite in recycled LDPE), which combine mechanical reinforcement, reduced water absorption, and improved flame retardancy [234,235,236]. Andrusyk et al. provide evidence for WPCs made from hot-water-extracted wood, showing that hemicellulose extraction can improve mechanical properties while maintaining flame performance [237]. Overall, these studies aim to increase the strength and stiffness of natural fiber and hybrid biocomposites through fiber selection, alignment, chemical treatment, and environmentally friendly or biodegradable matrices, emphasizing their role as “Biobased Fibre Composites Mechanics”.

Cluster #9 is led by the seminal review of Saheb and Jog on natural fiber–polymer composites, which already highlights moisture susceptibility and the need for improved durability [238]. Gilman and colleagues employed cone calorimetry and microscopy to investigate fire-retardant systems, focusing on the mechanistic understanding of flame retardancy embodied in hydrotalcites, graphene, cellulose nanofibrils, and polymer-layered silicate systems in polypropylene and polystyrene [239,240]. Haurie et al. investigated the thermal stability, limiting oxygen index (LOI), and cone calorimeter results of halogen-free minerals by analyzing the combination of magnesium hydroxide, aluminum hydroxide, and montmorillonite in LDPE/EVA blends and their flame-retardant thermocompositions [241,242]. This collection of publications demonstrates an ongoing research focus on the LOI, heat release rate, and char-residue stability in flame-retardant systems comprising magnesium and nanoclay. A precise explanation of co-citation encompasses studies on the design and evaluation of flame-retardant solutions for polyolefins and wood-plastic composite matrices, titled “Halogen-Free FR Minerals and Nanocomposites”.

Cluster #10 relates to the critical review published by Fu and colleagues on polymer nanocomposites, as well as studies on CNT-modified PP/wood-flour WPCs and particulate–polymer composites. These provide a clear framework showing how nanofiller type, dispersion, particle size, and interfacial adhesion influence stiffness, strength, toughness, and flammability in nano- and micro-filled systems [243,244,245,246]. The work of Park and collaborators focuses on urea-formaldehyde resins and rice husk surface modification, illustrating how the crystallinity and domain structure of cured UF, along with the surface chemistry of rice husks treated with MAPP or silane (characterized by XPS), affect hydrolytic stability, formaldehyde emission, particleboard performance, and interfacial bonding in wood-thermoplastic composites. Szego mill compounding demonstrates a continuous method for blending wood fiber with thermoplastics [247,248,249,250]. Wang et al.’s recent study on microwave-assisted acetic acid pretreatment of poplar for high-performance PE laminates further expands this interphase-engineering approach to glue-free wood–plastic laminates with very high strength and good water resistance [251]. Collectively, these references form a cluster centered on nanofillers, particulate reinforcement, and chemically tailored interfaces in wood–polymer systems, which is why the label “Nanofillers and Particulate Reinforcement” is fitting.

Cluster #11 builds upon the comprehensive works of Zhang and collaborators on thermoplastic starch (TPS/PLA blends with chain extenders, surface-modified wood flour, and nanofiber-reinforced polyolefin composites [252,253,254,255,256]. These studies repeatedly address glass transition, moisture sorption, tensile performance, and interfacial adhesion in bioplastic matrices. The series of Sanadi et al. research papers in this cluster again emphasizes lignocellulosic thermoplastics and agro-fiber reinforcements, but here specifically in the context of renewable agricultural fibers and the design of bio-based thermoplastic alloys [257,258,259,260,261]. Garcia et al.’s investigations on heat-treated fibers and MDF panels link thermal modification to surface energy, wetting, dimensional stability, and mechanical properties [262,263]. The shared focus on TPS/PLA bioplastic matrices combined with chemically or thermally modified lignocellulosic fibers as reinforcements, therefore, makes “Bioplastic Matrices and Modified Fibres” an appropriate and well-justified label for this cluster.

Cluster #12 is driven by Meyer and colleagues’ work, which explicitly explores wood impregnated with vinyl monomers and subsequent in situ polymerization within lumens and cell walls to create wood–polymer materials with improved hardness, abrasion resistance, dimensional stability, and strength, along with their industrial uses [264,265,266,267]. Additionally, Ibach and co-workers, in the “Specialty treatments” chapter, position wood–polymer systems among other permanent modification methods and concentrate on enhancing properties through non-wood additives such as resins, PEG, and acrylates [268]. Their research on bioactive monomers polymerized in situ directly aims to increase decay resistance at low polymer loading levels [269]. Glukhov et al.’s work on the radiation polymerization of vinyl monomers in wood also clearly belongs to the same family of in-wood polymerization processes [270]. Because of this strong and consistent focus on impregnation, in situ polymerization, and modification of solid wood—rather than particulate WPCs—the term “Impregnated Wood–Polymer Composites” is appropriate, effectively highlighting the cluster’s core methodology and application focus.

Cluster #13 focuses on phase change materials (PCMs) in building envelopes. Chang and collaborators numerically analyzed PCM/WPC roof modules, demonstrating that PCM phase-change temperature and thickness reduce roof surface temperatures and influence building energy performance [271]. Jamekhorshid et al. experimentally studied WPCs with microencapsulated PCM (MEPCM), proving leakage suppression, latent heat storage, and retention of mechanical properties [272]. Alshuraiaan et al. developed a computational model to optimize PCM layers in external walls in hot climates [273]. Co-citation thus clearly highlights the emerging field of PCM-integrated WPCs for thermal energy storage and building thermal regulation, thereby justifying the label “PCM-Integrated WPCs for Thermal Control,” which emphasizes building envelope thermal management.

Cluster #14 includes research by Ratanawilai and colleagues, which compared HDPE, LDPE, PP, PVC, and PS matrices for WPCs, evaluating mechanical performance and durability against natural weathering, while examining how wood species and particle size affect the mechanical and thermal properties of rPP composites [73,274]. García et al. demonstrate that combining fire retardants with light stabilizers creates a trade-off between auto-extinguishing behavior and color stability under outdoor conditions [275]. Wang et al. investigated ammonium polyphosphate, aluminum hydroxide, microencapsulated APP, and synthetic zeolite synergists, documenting the LOI, cone calorimeter parameters, char morphology, and hygrothermal properties of flame-retarded wood–PP and rice hull/HDPE composites [276,277,278,279,280]. When combined, these references present a unified narrative on the selection of polymer matrices and the arrangement of halogen-free flame-retardant systems, balancing mechanical properties, weathering durability, and fire protection in WPCs, as illustrated in the cluster label.

Finally, Cluster #15 is led by the work of Durmus and colleagues, mainly focused on measuring how wood fiber content, alkaline treatment, fiber size, and compatibilizer/wood ratio affect melt rheology and tensile behavior of PP–wood composites, using micromechanical models to connect improved interfacial adhesion with rheological signatures [281,282]. Additionally, Li et al.’s research on ultralong cellulose nanofibers from wood and cotton reveals significant improvements in flexural modulus, impact strength, and reduced thermal expansion of HDPE when CNF networks are well dispersed. It also compares CNF with NCC as nanofillers [283,284]. Liu and co-workers examine enzymatic hydrolysis of lignin as a multifunctional additive that enhances toughness and flowability, and develop HDPE/PA6 microfibrillar composites where coupling agents control fiber diameter and interfacial bonding; further studies compare various natural fiber types and coupling treatments in HDPE composites [285,286,287]. The main focus is on how nanocellulose, lignin, and microfibrillar structures influence rheology and reinforcement efficiency in highly filled polymer/wood systems, which explains the reference to nanofibers, lignin, and microfibrillar composites in the brief label “Nanofibres, Lignin and WPC Rheology”.

**Figure 8:**
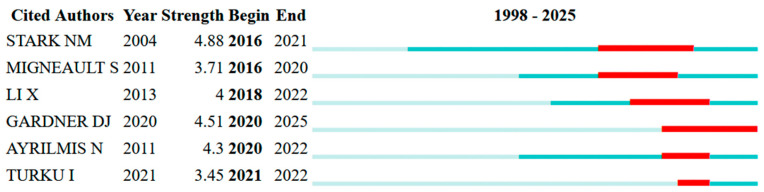
Top-cited authors with the strongest citation bursts.

Figure 8 shows the top six cited authors with the strongest citation bursts, indicating periods of rapid scholarly attention to WPCs. Stark NM from the USDA Forest Service, Forest Products Laboratory, has the strongest and longest burst (strength 4.88, 2016–2021). His work supports current understanding of the outdoor durability and fire performance of WPCs, including studies on heat-release behavior, fire-retardant additives in wood–polyethylene composites, and detailed FTIR/XPS analyses of weathered WPC surfaces and cap-stocked profiles. Migneault S from Université du Québec en Abitibi-Témiscamingue shows a strong burst from 2016–2020 (strength 3.71), driven by papers examining fiber surface chemistry, fiber origin, and fiber length distribution in relation to the mechanical strength, water uptake, and processing of HDPE-based WPCs. His studies illustrate how carbohydrate-rich surfaces and longer chemo-thermomechanical fibers enhance fiber–matrix bonding and composite strength. Li X from the Department of Agricultural and Bioresource Engineering at the University of Saskatchewan experienced a burst from 2018 to 2022 (strength 4.00), mainly connected to his widely cited review on chemical treatments of natural fibers for composite use and related work on BC/O-VMT nano-hybrid composites, bridging natural-fiber surface modification, and the performance of polymer composites used in WPC-related applications.

Gardner DJ from the University of Maine shows a recent, ongoing burst from 2020 to 2025 (strength 4.51). His work includes a ten-year field study on WPC durability in Chile and broad overviews of WPC technology and additive manufacturing with wood-based fillers, emphasizing long-term biological performance and new 3D-printing methods for wood-filled polymers. Ayrilmis N from Istanbul University-Cerrahpasa demonstrates a burst from 2020–2022 (strength 4.30), reflecting extensive research on formulation and fire-retardant design in WPCs and MDF—such as boron and phosphate FR systems, zinc-borate-modified composites, thermally treated fibers, and detailed studies of dimensional stability, surface quality, and creep. Turku I from FiberLaboratory, South-Eastern Finland University of Applied Sciences, has experienced a recent burst of activity from 2021–2022 (strength 3.45), focused on recycled-plastic WPCs, nanocomposites, and FR design, and on durability under accelerated weathering, including a comprehensive review of wood–plastic nanocomposites and work on FR-loaded co-extruded PP-based WPCs.

Overall, the bursts have been concentrated in the last decade and are led by authors focused on durability, fire performance, recycled matrices, fiber surface chemistry, and nanofillers, indicating that these topics are the most rapidly growing research areas in the WPC field.

Beyond the individually labeled clusters, the co-citation map also uncovers emerging research trends and hidden knowledge communities that exist at the intersections of established fields. Therefore, throughout the co-citation network, several interconnected research fronts can be identified, outlining the current intellectual landscape and emerging directions in WPC science.

A primary domain includes Clusters #3, #6, #9, and #14, which collectively address the thermal behavior, weathering, and fire performance of WPCs. These clusters demonstrate that WPC durability is now considered a combined issue of hygrothermal aging and fire safety, with matrix selection, FR formulation, and processing routes co-designed for exterior building applications.

The second, strongly interconnected domain concerns fiber surface chemistry, interfacial modification and hybrid reinforcement, primarily captured by Clusters #2, #5, and #7, but also intersecting with Cluster #11. Collectively, these clusters define a knowledge community in which the fiber/matrix interphase—rather than the bulk polymer alone—is the key design lever for mechanical performance and environmental resistance.

The third research front is centered on recycling, bio-based feedstocks, and advanced manufacturing, drawing mainly on Clusters #0, #6, #8, #10, and #15. Along with the additive-manufacturing work embedded in Cluster #6, these clusters point to a strong trend towards circular, resource-efficient WPCs produced not only by extrusion but also by flat-pressing and 3D printing.

Superimposed on these structural and processing developments is a coherent group of clusters devoted to nanostructured and multifunctional WPCs. Besides the nanofiller-intensive Clusters #3, #4, #9, #10, and #15, which investigate CNTs, nanoclays, mineral nanoparticles, and cellulose nanofibers, Cluster #4 (Functional Nanofillers and Hybrid WPCs) emphasizes functional interphases (e.g., antibacterial, conductive, or thermally conductive) in PVC and polyolefin systems. Across these clusters, the field is moving from simple stiffness enhancement towards multi-functional composites in which rheology, thermal expansion, barrier properties, and flame retardancy are tuned simultaneously through nano- and micro-scale structuring.

Finally, Clusters #12 and #13 represent more specialized, but clearly delineated, communities that extend WPC concepts beyond particulate composites. Although small in size, these clusters connect back to the main durability and fire-safety clusters, since both wood–polymer impregnates and PCM-integrated systems must meet stringent requirements for hygrothermal and fire performance.

When viewed together, the numbered clusters reveal an integrated research landscape where WPC science is transitioning from basic formulation work to durable, fire-safe, circular, and multifunctional wood-based polymer composites. This landscape features clear thematic communities focused on durability and fire, interfacial engineering, recycled or biobased feedstocks, nanostructuring, and advanced wood–polymer modification for building applications.

### 3.3. Co-Citation Analysis of Authors—RQ3

To identify the authors who have most strongly influenced the current review literature on WPCs, an analysis of author co-citation was conducted for 2020–2025. The chosen time frame is limited because the number of WPC-related reviews increases significantly after 2020, indicating a shift toward sustainability-focused composite development, thermomechanical optimization, and circular materials research. Limiting the analysis to this recent period allows for a targeted view of the authors currently shaping the field’s intellectual landscape, rather than those whose influence comes from earlier stages of development.

**Figure 9:**
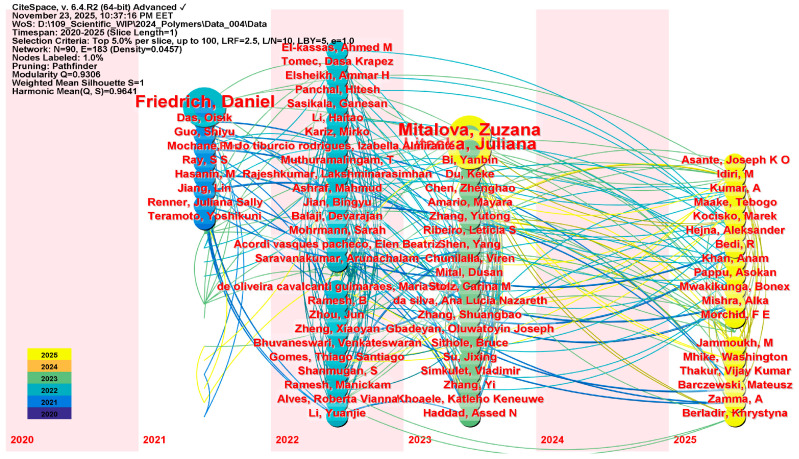
CiteSpace visualization of author co-citation timeline map (2020–2025).

Figure 9 shows the co-authorship of selected authors from 2020 to 2025, presenting a time-aligned co-citation diagram that reveals a complex intellectual network featuring four main authors, each contributing distinct themes and occupying unique positions within the citation network.

To start with, the left side of the timeline is mainly defined by Daniel Friedrich and colleagues, with a key, persistent node indicating the strongest and most ongoing co-citation over the years. Friedrich’s studies on thermoplastic molding by hot pressing [288], eco-labeled wood-plastic composites (WPCs) and their welfare effects [289], and their shape features [290] lay the conceptual and methodological groundwork for later research. From all these research studies, the authors gained knowledge of thermal conductivity, heat storage and retention, and the practicality and sustainability of hot-pressing manufacturing processes. As a result, Friedrich and his team could be seen as foundational stabilizers within the co-citation network, anchoring discussions on the thermomechanics and environmental sustainability of WPCs.

The subsequent dense central citation corridor (2021–2023) highlights the integrative contribution of Zuzana Mitaľová and her colleagues, recognized for their concise series of reviews on WPC matrices, components, and production technologies [10,42,291], as well as for their extensive citations across various fields. Their research on polymer matrix selection, filler-matrix interactions, additive functions, and production techniques such as extrusion, injection molding, resin transfer molding, and fused deposition modeling, builds a comprehensive body of knowledge. Therefore, it could be argued that Mitaľová and her collaborators serve as knowledge integrators, synthesizing the fundamentals of materials science with modern processing technologies and facilitating the coexistence of primary and auxiliary research.

Third, between 2022 and 2023, there is a notable concentration of authors whose work forms the core of the WPC review literature. It includes Elsheikh et al. [38], who synthesized pre-processing treatments, manufacturing methods, and recyclability assessments; Krapež Tomec et al. [35], who evaluated the use of wood fillers in additive manufacturing and 4D printing contexts; as well as Rodrigues et al. [292], who examined plastic lumber production as a reliable WPC-related approach for sustainable materials management in Brazil. These authors provide reviews that integrate advanced fabrication technologies, circular economy principles, and process optimization, thereby advancing the current technical development of WPC scholarship.

Fourth, several authors act as performance and application specialists, expanding the field into new functional and environmental areas. Jian et al. [39] focused on flexural performance, strengthening strategies, and mechanical optimization. Kumar et al. [293] studied the durability and mechanical behavior of composites that combine recycled PET matrices with natural fibers. Maake et al. [294] reviewed fire-retardant wood–polymer–rubber composites for built-environment applications. Morchid et al. [43] examined the incorporation of phase change materials (PCMs) to improve thermal performance, while Hejna et al. [295] proposed a paradigm shift by intentionally exceeding the temperature threshold through controlled processing degradation to reveal new functionalities of WPCs. These authors, positioned near the latter part of the period (2024–2025), are pioneering innovators advancing the research on WPCs in fire safety, thermal regulation, high-temperature production, and building energy consumption.

The co-citation timeline thoroughly illustrates a diverse range of essential foundational contributors to the literature on WPCs. Key researchers include stabilizers such as Friedrich, integrators such as Mitaľová, methodological pioneers such as Elsheikh, Tomec, and Rodrigues, and application experts such as Jian, Kumar, Maake, and Morchid. Concerning RQ3, the findings show that the most influential authors are those whose work displays high co-citation counts and significant structural influence, serving as core elements that connect different areas, develop new methods, and advance the field into new technological and application domains. This trend indicates an evolutionary stage within the research area. This suggests that the field is advancing in the development of sophisticated processing techniques, improved material properties, and the design of sustainable composites.

### 3.4. Co-Occurrence of Keywords—RQ4

To identify the leading and emerging research themes shaping current wood–plastic composite (WPC) scholarship, a keyword co-occurrence analysis was performed in CiteSpace using keywords that were extracted, normalized, and formatted according to the proposed Methodological Framework for Key Terms Mapping (MFKTM), as described in Section 2. Therefore, to address the underlying RQ4, the node type “Keywords” was selected in CiteSpace to identify thematic clusters and their intellectual connections.

**Figure 10:**
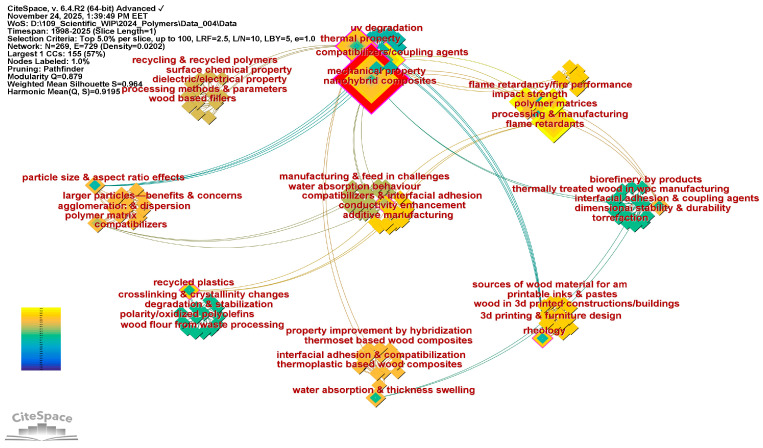
Keyword co-occurrence network for the WPC review corpus (CiteSpace).

Figure 10 shows the raw keyword co-occurrence network, where each node represents a unique keyword from the WPC-related review articles and each link indicates co-occurrence within the same publication. The network displays a dense web of intellectual connections, dominated by high-frequency terms such as mechanical property, processing and manufacturing, thermal property, compatibilizers/coupling agents, and impact strength.

The coloring of nodes illustrates the temporal distribution of co-occurrence activity from 1998 to 2025, showing how certain concepts—such as nanohybrid composites, UV degradation, and recycled plastics—become more prominent over time. The network’s main hotspot focuses on performance-related terms (e.g., mechanical properties, thermal properties), underscoring that optimizing structural, thermal, and interfacial behavior remains the most consistently discussed theme in the review studies on WPCs.

The peripheral groups of keywords highlight more specialized or emerging areas of research, such as printable inks, torrefaction, dielectric properties, particle size, additive manufacturing, and biorefinery by-products. These peripheral yet cohesive clusters show the growth of diverse subfields within the broader landscape of WPCs.

The citation burst analysis reveals “mechanical property” as the only keyword showing a strong and lasting citation burst during 2023–2025, with a strength of 3.85. It suggests that, despite the diversification of wood–plastic composite research, there has been a recent intensification of studies examining mechanical performance—such as stiffness, strength, durability, and interfacial behavior. The sharp increase in citations starting in 2023 indicates that optimizing mechanical properties remains a key focus in the current review literature on WPCs, reflecting ongoing challenges and rising interest in developing structurally reliable, application-ready composite materials.

**Figure 11:**
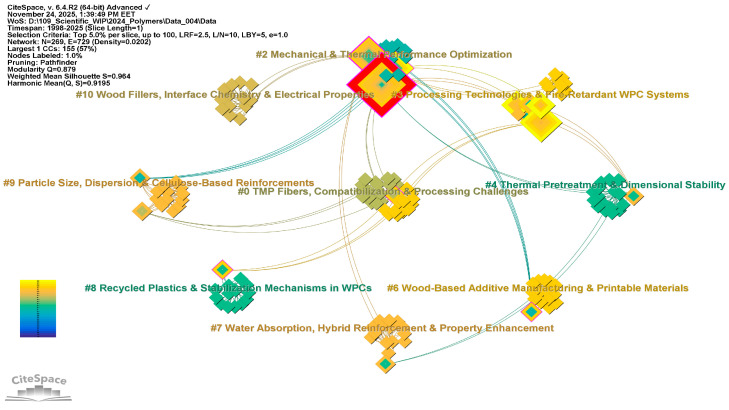
Clustered keyword co-occurrence network of WPC review literature (CiteSpace).

Figure 11 shows the clustered co-occurrence network of keywords, where CiteSpace’s clustering algorithm grouped the nodes into nine main thematic clusters that represent several clear research directions.

As shown graphically in the figure, the clusters display both topical proximity and a developmental sequence. On one hand, the large, centrally located clusters represent longstanding foundational themes, such as mechanical and thermal performance optimization, compatibilization, and processing challenges. Clusters situated toward the edges—such as wood-based additive manufacturing, recycled plastics and stabilization, and cellulose-based reinforcements—represent newer or rapidly growing themes that are increasingly shaping current research directions.

The temporal coloring of links illustrates that the network expands outward over time, with early research on WPCs focused on fundamental interface and mechanical behavior and later research branching into sustainability-driven, materials-innovation, and digital-manufacturing domains.

As shown below, the nine clusters described above provide the structural basis for understanding both the primary and emerging research themes that characterize today’s scholarship on WPCs.

The main themes in the review investigations on WPCs reflect longstanding concerns that have shaped the field for over 20 years. They are based on the fundamental relationship between materials composition, processing options, and property development, and are prominently featured across Clusters #0, #2, #3, and #4.

An example of the ongoing emphasis on interfacial design and compatibilization strategies is Cluster #0, which focuses on interfacial adhesion, the effectiveness of compatibilizers, and fiber–polymer compatibility. TMP fiber illustrates this well, and its intricate structure and tendency to absorb water make it both fascinating and challenging in reinforcement applications. Nevertheless, these interactions demand advanced methods for coupling and adjustments to processing parameters. Issues related to feed-in at an industrial level affect mechanical reliability, making this topic practically significant rather than merely scientifically interesting.

Cluster #2 reinforces the materials-focused foundation of WPCs by emphasizing the optimization of mechanical and thermal performance. In this context, the review studies highlight that improved reinforcement, nanofiller integration, and matrix modification are crucial for developing stable, high-performance composites. The use of terms such as UV degradation, nanohybrid composites, and thermal properties reflects a mature, engineering-driven effort to enhance composite performance for long-term outdoor structural applications.

Another leading thematic current concerns the integration of processing technologies with performance outcomes, represented by Cluster #3, since the involved review studies consistently demonstrate that the processing decisions—extrusion, injection molding, or mixing strategies—have direct consequences for property development. At the same time, the strong flame-retardant and impact-strength performance indicate that WPCs continue to expand into safety-critical applications. As a matter of fact, this reinforces the idea that WPCs are not only material alternatives but contenders for fire-safe panels, façade systems, and structural components.

Cluster #4 introduces an additional aspect by emphasizing thermal pretreatments and dimensional stability. The recurring themes of torrefaction, thermally treated wood, and biorefinery by-products indicate a growing interest in how chemical and structural modifications of lignocellulosic fillers affect swelling, durability, and interfacial properties. Therefore, it reflects a broader move toward resource-efficient material enhancement: improving performance not by sourcing new fibers but by better utilizing existing biomass streams.

The overlap of themes shows a strong research area that focuses on both technical accuracy and effectiveness. Together, they build a solid base for the discipline from which new research paths regularly develop. The main clusters keep the sector stable, while emerging topics indicate a clear move toward innovations in sustainability, functional material design, and digital manufacturing. The themes highlighted in Clusters #6, #7, #8, #9, and #10 represent the cutting edge of WPC research.

An emerging trend in the field is the integration of wood into additive manufacturing. Cluster #6 illustrates the evolution of research from combining wood flour with polymers to the creation of rheologically optimized printable inks for 3D and 4D printing. The network addresses printable pastes, additive manufacturing structures, and designs that can change shape. All of these emphasize the need to shift perceptions of WPCs from bulk materials to digitally fabricated materials with modified geometries.

Cluster #7 emphasizes a progressive focus: the interaction between hybrid reinforcement schemes and water-related deterioration. Key terms such as water absorption, thickness swelling, hybridization, and interfacial adhesion reflect a growing awareness of the need to design WPCs for moisture-prone environments. In fact, researchers are increasingly recognizing environmental durability, achieved through composite fibers or enhanced interface chemistry, as a crucial factor for wider adoption across the construction, maritime, and exterior design sectors.

In Cluster #8, which emphasizes maintaining plastic recycling and stabilization techniques, the WPC materials are recognized as a fundamental mechanized technology capable of converting waste streams—especially oxidized or degraded polyolefins—into high-value composites. The processes of degradation, stabilization, crosslinking, and variations in crystallinity indicate that understanding polymers’ recycling history and how stabilizers or compatibilizers can help prevent performance decline should be key research areas. This theme links research and innovation on WPCs directly to the global circular economy.

In Cluster #9, the focus shifts to particle size, dispersion traits, and cellulose-derived reinforcement, highlighting significant progress in materials science toward micro- and nanoscale uses. The references on agglomeration, aspect ratio, compatibilizers, and polymer matrix effects indicate that lignocellulosic additives are the primary focus of this cluster. Instead of replacing fibers, recent research has focused on using cellulose microstructures to enhance rheological, thermal, and mechanical performance.

Finally, Cluster #10 explores an additional versatile theme that combines wood additives and interface chemistry with dielectric and electrical properties. Regarding dielectric and electrical behavior, recycling, and processing, there is growing interest in WPCs as functional composites, likely for use in sensors, insulators, and electronic applications. This cluster probably represents the merging of polymer science, materials chemistry, and electrical engineering, which is an unexpected but potentially beneficial direction.

Overall, the nine clusters serve as evidence of a region undergoing substantial change. The main topics continue to advance the interaction among materials, interfaces, and processing, consistently aiming to produce dependable, high-performance WPCs. At the same time, several new clusters are becoming essential milestones for the subject, going beyond its usual limits to include sustainable materials engineering, digital manufacturing, functional composites, and circular resource efficiency.

### 3.5. Timeline View of Co-Cited Keywords—RQ5

**Figure 12:**
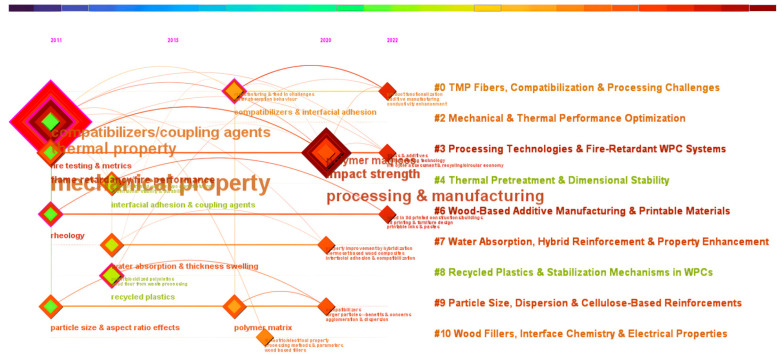
Timeline visualization of co-cited keywords in the WPC review corpus (CiteSpace).

Figure 12 shows a timeline visualization of how each thematic subgroup in the research on wood-plastic composite (WPC) materials has changed over time. It highlights their grouping, merging, and splitting. The nine theme clusters identified by keyword co-occurrence analysis are shown in the bands. The node’s color indicates the year with the most citations. History shows that a specialized field of study has shifted from focusing primarily on traditional performance measurements to encompassing a wide range of modern technologies and sustainability concerns.

The largest nodes are linked to Cluster #2, which centers on mechanical and thermal performance optimization. Terms like mechanical properties, thermal properties, compatibilizers/coupling agents, and interfacial adhesion became significant in the field after 2011. The size and prominence of the node show that improving the composite’s performance has always been a key goal in WPC research. Based on current activity, the materials and interfaces area will likely focus on additives, coupling agents, polymer matrices, and processing techniques to improve the composite’s mechanical strength and thermal stability.

From 2017 to 2021, the timeline shows the development of clusters focused on processing technology and fire-retardant performance (Cluster #3) as well as on thermal pretreatment and dimensional stability (Cluster #4). Key terms such as processing and manufacturing, polymer matrices, impact strength, flame retardancy, thermally treated wood, and dimensional stability indicate that research on WPCs is advancing to meet application needs in building materials, furniture design, transportation, and outdoor structures. However, during this period, one may observe a shift from merely optimizing materials to preparing them for practical use, enhancing fire safety, dimensional stability, and compatibility with industrial processing systems.

Beginning in 2020, the timeline visualization highlights another key turning point in research on WPCs—shifting from minor performance and processing improvements to a broader, future-focused approach driven by sustainability, resource circularity, and digital fabrication technologies. This period shows a notable increase in thematic diversity, with several new clusters emerging and developing over time. They collectively represent a field beyond conventional composite formulations, entering an area where environmental responsibility, the utilization of multifunctional materials, and advanced manufacturing techniques converge. A key advancement during this period is the emergence of wood-based additive manufacturing (Cluster #6). Part of these studies involves integrating WPC materials with 3D and 4D printing technologies to enable the creation of lightweight, digitally produced components with complex shapes. The growing association of terms such as rheology, printable ink, 3D printing, and printed structures/buildings suggests that WPCs are not just bulk materials for extrusion, but are instead a new engineered feedstock for digital fabrication. This perspective reflects the broader global trend toward construction automation, mass customization, and efficient production with minimal waste, further supporting the use of WPCs in sustainable additive manufacturing.

The timeline also shows a growing focus on recycled plastics and stabilization methods (Cluster #8). The appearance of terms like recycled plastics, oxidized polyolefins, degradation, and stabilization, along with changes in crystallinity, indicates a significant shift in environmental research. Therefore, as interest in the circular economy rises, WPC materials are increasingly seen as an essential means of reusing plastic waste streams. The scientific discussion goes beyond replacing virgin polymers to address complex issues like material aging, oxidation behavior, compatibilization needs, and long-term stability. Such progress highlights a closer link between the science and engineering of WPCs on the one hand, and the global environmental goals on the other.

An additional significant factor relates to the advances in research on cellulose-based reinforcements and particle engineering (Cluster #9). Growing interest in micro- and nanocellulose indicates a shift toward bio-based enhancements, where particle size, morphology, and dispersion control are crucial for customizing the properties of WPCs. Key terms related to agglomeration, aspect ratio, and compatibilizers increasingly define this field, indicating that researchers are exploring ways to leverage cellulose’s high aspect ratio, renewability, and inherent strength to improve the performance of WPC materials while reducing their environmental impact.

In addition, water-resistance engineering and hybrid reinforcement techniques (Cluster #7) have gained prominence since 2022. As the use of WPCs extends to outdoor, marine, and moisture-laden environments, persistent concerns about water absorption, thickness swelling, hybridization, and interfacial adhesion underscore ongoing efforts to improve durability. In fact, this area of research demonstrates how practical performance difficulties are motivating the design of innovative materials, in which hybrid fillers or altered interfaces mitigate moisture sensitivity.

When considered together, all these clusters exemplify a research environment that is systematically broadening its scope. Recent research increasingly emphasizes material recyclability, bio-based reinforcements, moisture- and durability-engineering, and the integration of digital production, rather than focusing mainly on mechanical and thermal optimization, which have already been developed. Such a diversity of themes indicates that WPCs are evolving from a traditional category of polymer composites into versatile, sustainability-focused, and technologically advanced material systems. For this reason, the subject now encompasses not only the current state of WPCs but also their potential development within larger environmental and technological contexts.

### 3.6. Precision over Proxy: The Methodological Framework for Key Terms Mapping (MFKTM)

Adopting the proposed Methodological Framework for Key Terms Mapping (MFKTM) significantly improved the topical accuracy of our analysis compared to using Keywords Plus or even Author Keywords. By extracting terms from different sections of each manuscript and then standardizing synonyms into canonical labels, the framework based the vocabulary on what the investigated publications “do” (i.e., methods, results, and applications), rather than on citation-based proxies. This section-aware, canonicalized term set proved to be both more accurate and more comparable across studies, enabling more apparent clustering and more precise trend analysis. Since each term links back to its source section, the map is auditable and reproducible, thereby enhancing the interpretability of the results.

The method offered additional benefits. Canonicalization fixed spelling and terminology inconsistencies (such as molding/moulding; fiber/fibre; WPC/WPCs), improved cross-document matching, and prevented keyword sprawl by standardizing item and quantity formats. In practice, curated, semicolon-separated term strings can be used directly in subsequent analyses (e.g., co-occurrence networks and topic clustering) without additional cleaning, thereby enhancing analysis efficiency.

These advantages come with trade-offs. Full-text extraction, sectioned retrieval, and manual synonym mapping are more labor-intensive and may not easily scale to large corpora; evaluating what is considered “salient” also involves some subjectivity. To address these issues, we combined curation with lightweight Natural Language Processing (NLP) pre-screening, used controlled, versioned vocabulary for canonical terms, and kept an audit trail to support inter-annotator checks.

The advantages surpass the disadvantages, as MFKTM offers superior topical accuracy, enhanced comparability, and improved traceability relative to standard keyword fields, while the necessary processing effort can be effectively managed through a hybrid workflow and continuous maintenance of the controlled vocabulary.

## 4. Conclusions

The co-citation analysis of references (RQ1) shows that WPC research is organized around three closely connected intellectual pillars rather than separate topics. First, material reinforcement and interfacial engineering form the main foundation of the field, with highly co-cited works showing that fiber type, morphology, and surface chemistry—along with suitable coupling agents—control stiffness, strength, dimensional stability, and long-term performance. Second, processing methods and structure–property relationships stand out as a distinct yet strongly linked pillar, where extrusion versus injection molding, fiber size preparation, rheology, and microstructural development are shown to “imprint” performance during manufacturing. Third, a quickly growing cluster of studies on sustainability, recycling, fire safety, thermal pretreatment, and PCM-based thermal management indicates a strategic shift toward environmentally friendly, durable, and multifunctional WPC systems.

The clustering of co-cited authors (RQ2) shows that modern WPC research is organized into 16 cohesive knowledge communities rather than isolated topics. These clusters combine into a few main areas: (i) material reinforcement and interfacial engineering, focusing on natural and synthetic fibers, coupling agents, and fiber surface chemistry; (ii) durability, weathering, and fire performance, where hygrothermal aging, matrix selection, and halogen-free flame-retardant design are seen as interconnected challenges; and (iii) sustainability-driven development, including recycled and agro-based fillers, bioplastic matrices, and advanced manufacturing methods such as co-extrusion, flat-pressing, and 3D printing. These are strongly linked clusters on nanostructured and multifunctional WPCs, covering nanoclays, CNTs, mineral nanoparticles, nanocellulose, and PCM-integrated systems for thermal management, as well as solid wood–polymer impregnates. Citation-burst analysis highlights that the most active fields are durability and fire safety, recycled and waste-derived matrices, fiber surface modification, and nano-enabled flame-retardant or functional interfaces.

The co-citation analysis of authors (2020–2025) shows that the current WPC review literature is primarily shaped by a structured network of intellectual roles rather than by isolated contributions. Several leading authors serve as foundational stabilizers, offering core thermomechanical and sustainability frameworks that many subsequent reviews build upon. Meanwhile, others act as integrative connectors, linking polymer selection, filler interactions, and processing technologies across multiple thematic areas. A group of methodological innovators advances the integration of advanced fabrication techniques, recyclability assessments, and circular economy principles. At the forefront of research, application-focused specialists expand WPCs into fire safety, thermal regulation, high-temperature processing, and building-energy applications.

The keyword co-occurrence analysis (RQ4) indicates that the WPC review literature continues to focus on performance-related themes, with “mechanical property” being the only recent notable burst keyword. This fact confirms that stiffness, strength, and durability remain key research areas. Core clusters emphasize longstanding topics such as interfacial adhesion and compatibilization, improvements in mechanical and thermal performance, processing–structure–property relationships, and thermal pretreatments to enhance dimensional stability. New clusters also highlight emerging themes across integrated focus areas in sustainability and technological innovation. These include wood-based additive manufacturing (3D/4D printable inks), hybrid reinforcement systems, moisture-resistant WPCs, and tailored dielectric and functional properties. In addition, it is worth mentioning that topics such as torrefied biorefineries, cellulose derived from textiles and fillers, stabilization methods, bioplastics, and micro- and nano-cellulose reinforcements are gaining interest.

The timeline analysis of co-cited keywords (RQ5) indicates that from 2017 to 2021, research on WPCs has progressed comprehensively, emphasizing mechanical and thermal performance, compatibilization, and interface development, in line with improvements in processing optimization, fire-retardant performance, and thermal pretreatment to enhance dimensional stability. These advances have addressed the requirements for structural and exterior applications. Recent clusters identified since 2020 also illustrate the incorporation of sustainability- and technology-oriented frameworks within wood-based additive manufacturing. The use and stabilization of recycled plastics, along with micro- and nanocellulose reinforcements, are also part of a more hybrid approach. Additionally, growth has been seen in moisture systems, blockages, and aggressive environments.

Replacing citation-based fields with the MFKTM pipeline (i.e., multi-section term extraction plus synonym harmonization into a controlled, canonical vocabulary) produced a thematic map that is significantly more accurate, comparable, and auditable than one created from Keywords Plus or Author Keywords provided by the WoS database. The curated term sets are seamlessly incorporated into co-occurrence and clustering analyses without additional processing, thereby facilitating the interpretation of cross-study trends. This method improves precision regarding the subject, ensures uniform terminology throughout the primary documents, and facilitates tracing specific terms to their source segments, thereby supporting more effective analysis. The trade-off, however, involves increased curation effort and the potential for bias, which reduces scalability; yet, the benefits clearly outweigh the drawbacks. On the other hand, the benefits outweigh the costs, as the proposed MFKTM approach provides a reliable and reproducible basis for thematic mapping and is thus recommended for future literature syntheses across many other engineering fields.

## Figures and Tables

**Table 1:** The proposed keyword strategy for the WOS core collection database search.

Field Tag	Strategic Query	
Topic (i.e., Title + Abstract + Keywords)	“wood-polymer *” OR “wood polymer *” OR “wood-plastic *” OR “wood plastic *”	
	AND	“composite”
	AND	“review” OR “literature review” OR “systematic review” OR “scoping review” OR “state of the art” OR “bibliometric analysis”

**Table 2:** Summary of the 12 reference co-citation clusters identified in the WPC review literature.

Research Direction	Cluster Title	Cluster ID *
Material Reinforcement and Interfacial Engineering	Reinforcement Strategies and Mechanical Properties	#0
	Wood Flour–Polyolefin Composites and Interfacial Modification	#1
	Silicon-Based Surface Chemistry and Adhesion Enhancement	#3
	Multicomponent Polymer–Wood Systems and Compatibilization	#6
	Coupling Agents and Interfacial Engineering in Biocomposites	#18
Processing Approaches and Structure–Property Relationships	Processing Routes, Fiber Size Effects, and Composite Structure	#5
	Rheological Behavior and Processing Optimization	#9
Sustainability, Recycling, Fire Safety, and Thermal Enhancement	Thermal Pretreatment of Wood and Hygrothermal Stability	#2
	Biodegradable Polymer–Wood Composites and Fiber Morphology Effects	#7
	Wood Modification, Fire Resistance, and Thermochemical Stability	#16
	Manufacturing Techniques, Recyclability, and Fire-Retardant Performance	#17
	Thermal Energy Storage via PCM Integration	#19

* See Figure 4 and Figure 5.

## Data Availability

The bibliometric dataset (Web of Science export) and processed term lists used for the MFKTM analysis are openly available in FigShare at https://doi.org/10.6084/m9.figshare.30796598 (accessed on 28 November 2025).

## Supplementary Materials

The following supporting information can be downloaded at: https://www.mdpi.com/article/10.3390/polym18010063/s1, Table S1. PRISMA 2020 checklist.
