# Supplementary material for: A Meta-Synthesis of Review Studies on Wood–Polymer Composites: Mapping the Current Research Landscape
Source: Polymers (Basel). 2025 Dec 25;18(1):63. doi: 10.3390/polym18010063 (PMC12787367; doi:10.3390/polym18010063)
Supplement: Supplementary file 1 [file polymers-18-00063-s001.zip › polymers-4056980-supplementary.pdf]

Table S1. PRISMA 2020 checklist for the meta-synthesis of review studies on wood–polymer composites, indicating where each reporting item is addressed in the manuscript (source: https://www.prisma-statement.org/prisma-2020-statement).

Section and Topic	Item #	Checklist Item	Location Where the Item Is Reported
TITLE			
Title	1	Identify the report as a systematic review.	Title page: "A Meta-Synthesis of Review Studies on Wood–Polymer Composites: Mapping the Current Research Landscape" and "Type of the paper (Review)" at the top.
ABSTRACT			
Abstract	2	See the PRISMA 2020 for Abstracts checklist.	The following sentence is near the end of the abstract's methods part: "A structured search of the Web of Science Core Collection …".
INTRODUCTION			
Rationale	3	Describe the rationale for the review in the context of existing knowledge.	Introduction, first paragraphs (context of WPCs, applications, sustainability motivations), and later paragraphs explaining the need to consolidate fragmented review literature.
Objectives	4	Provide an explicit statement of the objective(s) or question(s) the review addresses.	End of Introduction, where RQ1–RQ5 are explicitly defined.
METHODS			
Eligibility criteria	5	Specify the inclusion and exclusion criteria for the review and how studies were grouped for the syntheses.	Section "2. Data source and methodology", paragraphs describing inclusion of review-type articles (narrative/systematic/scoping/bibliometric) focused on WPCs and exclusion of non-reviews or papers where WPCs were only peripheral.
Information sources	6	Specify all databases, registers, websites, organizations, reference lists, and other sources searched or consulted to identify studies. Specify the date when each source was last searched or consulted.	Same Section 2, "data collection … WoS Core Collection database", and note that all searches were completed by June 2025.
Search strategy	7	Present the full search strategies for all databases, registers, and websites, including filters and limits used.	Section 2 plus Table 1 ("The proposed keyword strategy for the WoS core collection database search"), with the exact Topic (TS) query string.
Selection process	8	Specify the methods used to decide whether a study met the inclusion criteria of the review, including how many reviewers screened each record and each report retrieved, whether they worked independently, and if applicable, details of automation tools used in the process.	Section 2 ("The screening process was carried out in two stages …", and ("Two reviewers independently assessed eligibility …").
Data collection process	9	Specify the methods used to collect data from reports, including how many reviewers collected data from each report, whether they	Section 2 ("Data extraction from the included articles was carried out …", and

		worked independently, any processes for obtaining or confirming data from study investigators, and if applicable, details of automation tools used in the process.	("Two reviewers independently checked the exported …").
Data items	10a	List and define all outcomes for which data were sought. Specify whether all results that were compatible with each outcome domain in each study were sought (e.g., for all measures, time points, analyses), and if not, the methods used to decide which results to collect.	RQ1–RQ5 define the "outcomes": co-citation structure, clusters, author roles, keyword themes, timeline evolution. Section 3 explains co-citation networks, author clusters, keyword co-occurrence, and timelines as the core analytic outputs.
	10b	List and define all other variables for which data were sought (e.g., participant and intervention characteristics, funding sources). Describe any assumptions made about any missing or unclear information.	Section 2 (WoS export fields, reference data, author names), and narrative parts of Results, where the characteristics of the included reviews (topics, types, time span, etc.) are discussed.
Study risk of bias assessment	11	Specify the methods used to assess risk of bias in the included studies, including details of the tool(s) used, how many reviewers assessed each study and whether they worked independently, and if applicable, details of automation tools used in the process.	Not applicable. Reason specified in Section 2 ("No formal risk-of-bias tool was applied because the study focuses on mapping the intellectual …).
Effect measures	12	Specify for each outcome the effect measure(s) (e.g., risk ratio, mean difference) used in the synthesis or presentation of results.	Not applicable. Reason specified in Section 1 ("… this review does not assess intervention effects, as the outcomes are based on network properties such as …).
Synthesis methods	13a	Describe the processes used to decide which studies were eligible for each synthesis (e.g., tabulating the study intervention characteristics and comparing against the planned groups for each synthesis (item #5)).	Section 2. Data source and methodology (CiteSpace network analysis; MFKTM) + MFKTM subsection.
	13b	Describe any methods required to prepare the data for presentation or synthesis, such as handling of missing summary statistics, or data conversions.	Not applicable—no statistical meta-analysis performed; synthesis is descriptive and network-based (Section 2).
	13c	Describe any methods used to tabulate or visually display results of individual studies and syntheses.	Not applicable—no heterogeneity analysis; no pooled effect estimates.
	13d	Describe any methods used to synthesize results and provide a rationale for the choice(s). If meta-analysis was performed, describe the model(s), method(s) to identify the presence and extent of statistical heterogeneity, and software package(s) used.	Not applicable—no sensitivity analyses; no quantitative pooling of effects.
	13e	Describe any methods used to explore possible causes of heterogeneity among study results (e.g., subgroup analysis, meta-regression).	
	13f	Describe any sensitivity analyses conducted to assess robustness of the synthesized results.	
Reporting bias assessment	14	Describe any methods used to assess risk of bias due to missing results in a synthesis (arising from reporting biases).	Not applicable—no publication-bias tests (e.g., funnel plots), as no quantitative effect sizes were pooled (see Section 2/Limitations in Discussion).

Certainty assessment	15	Describe any methods used to assess certainty (or confidence) in the body of evidence for an outcome.	Not applicable—no GRADE/certainty assessment; no body of intervention effect estimates was graded.
RESULTS			
Study selection	16a	Describe the results of the search and selection process, from the number of records identified in the search to the number of studies included in the review, ideally using a flow diagram.	Section 2. Data source and methodology, paragraphs 37–38 + Figure 2 (PRISMA flowchart).
	16b	Cite studies that might appear to meet the inclusion criteria, but which were excluded, and explain why they were excluded.	Section 2. Data source and methodology, bullet list of exclusion criteria (non-review; WPC not main focus).
Study characteristics	17	Cite each included study and present its characteristics.	Section 3. Results and Discussion (3.1–3.5), where the included reviews are described by topic, type, and cluster; Table 2 (co-citation clusters) and narrative around key reviews (e.g., Friedrich, Mitaľová, Elsheikh, etc.).
Risk of bias in studies	18	Present assessments of risk of bias for each included study.	Not applicable—no formal risk-of-bias assessment of individual reviews performed.
Results of individual studies	19	For all outcomes, present, for each study: (a) summary statistics for each group (where appropriate) and (b) an effect estimate and its precision (e.g., confidence/credible interval), ideally using structured tables or plots.	Not applicable—no individual intervention effect estimates; individual reviews are discussed qualitatively in Section 3.
Results of syntheses	20a	For each synthesis, briefly summarize the characteristics and risk of bias among contributing studies.	Section 3. Results and Discussion (Sections 3.1–3.5) + Section 4. Conclusions (summary of three pillars and clusters)
	20b	Present results of all statistical syntheses conducted. If meta-analysis was done, present for each summary estimate and its precision (e.g., confidence/credible interval) and measures of statistical heterogeneity. If comparing groups, describe the direction of the effect.	Not applicable—no quantitative synthesis or heterogeneity analysis.
	20c	Present results of all investigations of possible causes of heterogeneity among study results.	Not applicable—no sensitivity analyses conducted.
	20d	Present results of all sensitivity analyses conducted to assess the robustness of the synthesized results.	Not applicable—robustness not assessed by quantitative sensitivity analysis; findings interpreted qualitatively in Sections 3 and 4.
Reporting biases	21	Present assessments of risk of bias due to missing results (arising from reporting biases) for each synthesis assessed.	Not applicable—no formal assessment of reporting bias; potential selection bias from using WoS and English-only discussed in Limitations (Discussion).
Certainty of evidence	22	Present assessments of certainty (or confidence) in the body of evidence for each outcome assessed.	Not applicable—no GRADE/certainty assessment performed.
DISCUSSION			
Discussion	23a	Provide a general interpretation of the results in the context of other evidence.	Section 3. Results and Discussion (synthesis of pillars/clusters vs. prior WPC reviews) + Section 4. Conclusions.

	23b	Discuss any limitations of the evidence included in the review.	Section 3/early part of Section 4, where the coverage of WPC review literature and thematic biases are discussed; MFKTM limitations paragraph.
	23c	Discuss any limitations of the review processes used.	Section 2 and Discussion–remarks on single database (WoS Core Collection), English-language restriction, and manual nature of MFKTM curation (limitations paragraph).
	23d	Discuss implications of the results for practice, policy, and future research.	Section 3. Results and Discussion (future directions for WPCs) + Section 4. Conclusions (research gaps and recommended directions).
OTHER INFORMATION			
Registration and protocol	24a	Provide registration information for the review, including register name and registration number, or state that the review was not registered.	Section 2. Data source and methodology: “This review was not prospectively registered, and no separate protocol was prepared.”
	24b	Indicate where the review protocol can be accessed, or state that a protocol was not prepared.	Section 2. Data source and methodology: “This review was not prospectively registered, and no separate protocol was prepared.”
	24c	Describe and explain any amendments to information provided at registration or in the protocol.	Not applicable—no registered protocol to amend.
Support	25	Describe sources of financial or non-financial support for the review, and the role of the funders or sponsors in the review.	“Funding” section at the end.
Competing interests	26	Declare any competing interests of review authors.	“Conflicts of Interest” section at the end.
Availability of data, code, and other materials	27	Report which of the following are publicly available and where they can be found: template data collection forms; data extracted from included studies; data used for all analyses; analytic code; any other materials used in the review.	“Data Availability Statement” section at the end.
